# New Insights Into Cholinergic Neuron Diversity

**DOI:** 10.3389/fnmol.2019.00204

**Published:** 2019-08-27

**Authors:** Noorya Yasmin Ahmed, Rhys Knowles, Nathalie Dehorter

**Affiliations:** Eccles Institute of Neuroscience, John Curtin School of Medical Research, Australian National University, Canberra, ACT, Australia

**Keywords:** interneurons, acetylcholine, diversity, striatum, development

## Abstract

Cholinergic neurons comprise a small population of cells in the striatum but have fundamental roles in fine tuning brain function, and in the etiology of neurological and psychiatric disorders such as Parkinson’s disease (PD) or schizophrenia. The process of developmental cell specification underlying neuronal identity and function is an area of great current interest. There has been significant progress in identifying the developmental origins, commonalities in molecular markers, and physiological properties of the cholinergic neurons. Currently, we are aware of a number of key factors that promote cholinergic fate during development. However, the extent of cholinergic cell diversity is still largely underestimated. New insights into the biological basis of their specification indicate that cholinergic neurons may be far more diverse than previously thought. This review article, highlights the physiological features and the synaptic properties that segregate cholinergic cell subtypes. It provides an accurate picture of cholinergic cell diversity underlying their organization and function in neuronal networks. This review article, also discusses current challenges in deciphering the logic of the cholinergic cell heterogeneity that plays a fundamental role in the control of neural processes in health and disease.

## Introduction

Cholinergic neurons are highly integral for fine tuning brain function (for review, see Bonsi et al., 2011) and maintaining the excitation-inhibition balance within neural circuits (Zhou et al., 2017). Although cholinergic neurons are distributed in various discrete regions, they can project to almost all parts of the brain (Dautan et al., 2016). These cells release acetylcholine (ACh), which plays a crucial role in the regulation of sensory function (Minces et al., 2017; for review, see Ballinger et al., 2016), and actions (Bradfield et al., 2013; Markowitz et al., 2018). ACh also attunes motivation (Marche et al., 2017), behavioral flexibility (Aoki et al., 2015, 2018; Okada et al., 2018), and associative learning (Atallah et al., 2014; for review, see Yamanaka et al., 2018). A major population is found in the striatum, which contains the highest levels of ACh in the brain (Macintosh, 1941; for review, see Lim et al., 2014). Cholinergic neurons are also found in the basal forebrain, which is classically segregated into four main regions: the Medial Septal Nucleus (MSN), the vertical and horizontal limbs of the Diagonal Band of Broca (DB), and the Nucleus Basalis (NB) of Meynert. Within the brainstem, cholinergic neurons are found in the pedunculopontine nucleus (PPN) and the laterodorsal tegmentum (for review, see Mesulam et al., 1983). In addition to these groups, smaller cholinergic populations are located in the medial habenula (López et al., 2019), parabigeminal nucleus (Mufson et al., 1986), cerebral cortex (von Engelhardt et al., 2007), hypothalamus (Jeong et al., 2016), and olfactory bulb (Krosnowski et al., 2012).

**Figure 1:**
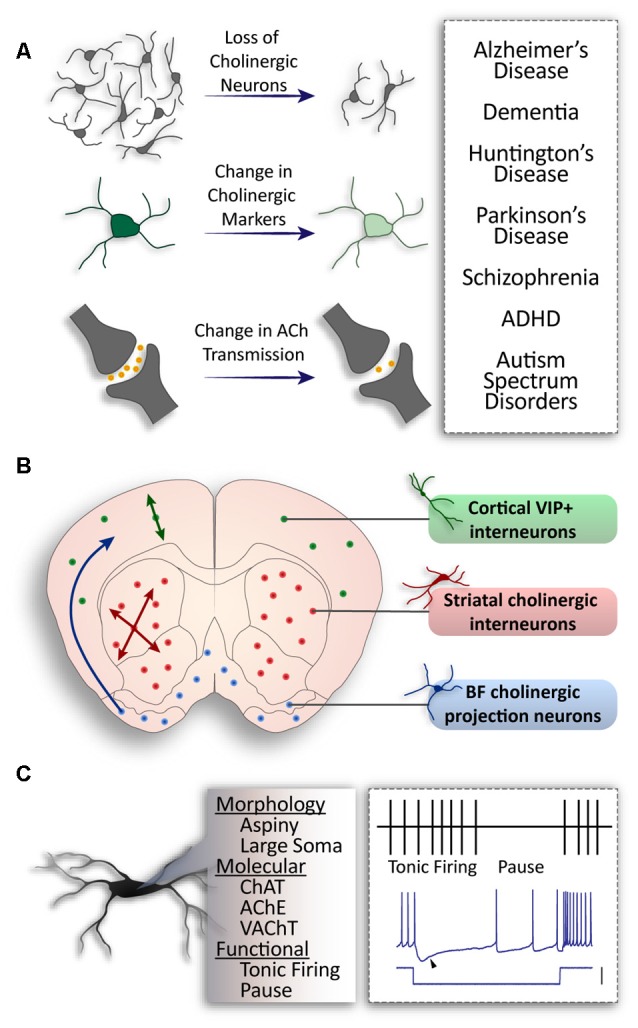
General populations of cholinergic neurons and alterations in disease. **(A)** Pathologies associated with cholinergic alteration. **(B)** Three major populations of cholinergic neurons in the brain. Basal forebrain neurons project to higher structures (blue); striatal interneurons project locally throughout the striatum (red); cortical interneurons co-expressing Vasoactive Intestinal Peptide (VIP) and Choline Acetyltransferase (ChAT) project within cortical layers (green). **(C)** Common properties of cholinergic neurons: aspiny dendrites and a large soma; expression of ChAT, Acetylcholine Esterase (AChE), Vesicular Acetylcholine Transporter (VAChT) expression; tonic firing patterns with a distinct pause *in vivo*, and a sag in membrane voltage (arrow) in response to hyperpolarizing current (scale: 100 pA).

It has been shown that changes in the activity of striatal cholinergic interneurons (CINs) play a critical role in motor control (Bordia et al., 2016), as well as behavioral flexibility (Okada et al., 2014), memory (Albert-Gascó et al., 2017), and social behavior (Martos et al., 2017). Alterations to the cholinergic system can lead to severe dysfunction of neuronal circuits (Figure 1A). For example, cholinergic neuron loss from the forebrain causes cognitive deficits associated with Parkinson’s (PD; for review, see Pepeu and Grazia Giovannini, 2017) and Alzheimer’s Disease (AD; for review, see Hampel et al., 2018). In the striatum, a decrease in cholinergic markers is a phenotypic consequence of Parkinson’s (Maurice et al., 2015; Ztaou et al., 2016) and Huntington’s diseases (HD; Smith et al., 2006). Cholinergic neuron populations are also linked to neuropsychiatric and neurodevelopmental pathologies. For instance, the activity of CINs through the expression and function of the hyperpolarization-activated cyclic nucleotide-gated channel 2 (HCN2) is decreased in the Nucleus Accumbens (NAc) in stress and depression (Cheng et al., 2019). There is also an established link between the cholinergic system and autism spectrum disorders, whereby autism is associated with decreased cholinergic tone and reduced neurite arborization (Nagy et al., 2017). Genetic alterations of the choline transporter cause attention deficit hyperactivity disorder (ADHD) and result in the promotion of ACh synthesis (English et al., 2009). Moreover, elevating ACh levels can relieve cognitive and social symptoms in a mouse model of autism (Karvat and Kimchi, 2014). Aberrant cholinergic signaling has also been reported in schizophrenia (for review, see Terry, 2008; Higley and Picciotto, 2014). However, there is still much debate over the mechanisms underlying cholinergic dysfunction in this disease (for review, see Hyde and Crook, 2001; Scarr et al., 2013). Considering that cholinergic dysfunction is associated with a diverse group of diseases, it is important to understand the functional intricacies of these cells.

Complex and dense cholinergic innervation is achieved in the brain from two categories of cholinergic neurons: the CINs—which synapse locally within a brain structure, and projecting cholinergic neurons—which send their axons to other structures (for review, see Allaway and Machold, 2017). CINs are present in a number of structures where they are important for local modulation. For example, cortical CINs, known for their expression of Vasoactive Intestinal Peptide (VIP), comprise approximately 12% of cortical inhibitory neurons (von Engelhardt et al., 2007; for review, see Rudy et al., 2011). They are primarily bi-tufted and found in layer 2/3 of the cortex (von Engelhardt et al., 2007). However, bipolar and multipolar CINs are also present and populate lower cortical layers (Li et al., 2018). On the contrary, striatal CINs are much larger, with extensive axonal fields (for review, see Lim et al., 2014) to control the output of the striatal projection neurons (SPNs). Projecting cholinergic neurons are found in the basal forebrain, hypothalamus and medial habenula (Li et al., 2018) and send long-range axonal projections to various cortical and subcortical regions (Figure 1B). Moreover, cholinergic neurons found in the PPN project to the basal ganglia, thalamus, and hypothalamus (for review, see Mena-Segovia and Bolam, 2017; French and Muthusamy, 2018).

In addition to their different projection patterns, cholinergic neurons can also be characterized by their firing properties. PPN cholinergic neurons display phasic and short-latency responses and provide a fast and transient response to sensory events (Petzold et al., 2015), whereas CINs of the striatum switch from tonic firing to a transient pause (Figure 1C; Shimo and Hikosaka, 2001; Atallah et al., 2014). CINs precisely control striatal output (Goldberg and Reynolds, 2011; Faust et al., 2015; Zucca et al., 2018) by inhibiting the firing of SPNs *via* neuropeptide-Y expressing inhibitory interneurons (English et al., 2011). They also drive spontaneous activation of SPNs *via* muscarinic (Mamaligas and Ford, 2016) and glutamatergic transmission (Higley et al., 2011). The spontaneous firing patterns of striatal CINs are sculpted by the widely studied delayed rectifier (IKr) and hyperpolarized activated current (Ih; Oswald et al., 2009). Calcium-dependent potassium conductances also give rise to prominent afterhyperpolarizations, and striatal CIN firing includes two periodic patterns: single spiking and rhythmic bursting (Bennett et al., 2000; Goldberg and Wilson, 2005; Figure 1C).

In addition to physiological characteristics, cholinergic neurons share genetic commonalities. The expression of choline acetyl-transferase (ChAT) and acetylcholine esterase (AChE) are markers of all cholinergic neurons, as they are involved in the synthesis and degradation of ACh respectively (Taylor and Brown, 1999). During development, they express common transcription factors that are conserved across neuronal populations. For instance, the homeobox-encoding Nkx2.1 transcription factor is expressed in many sites of neurogenesis within the developing brain such as the Medial Ganglionic Eminence (MGE), preoptic area (POA), septal neuroepithelium (SE). It has been shown that most cholinergic neurons arise from Nkx2.1^+^ progenitors (for review, see Allaway and Machold, 2017), and that cells expressing Nkx2.1 that upregulate the LIM homeobox transcription factors Islet-1 (Isl1) and Lhx8 become cholinergic (Zhao et al., 2003; Cho et al., 2014). As these transcription factors are expressed early during development, their co-expression is likely to be one of the earliest traits of cholinergic fated neurons (Fragkouli et al., 2009). Moreover, a loss of Nkx2.1 expression during development halts the maturation and specification of cholinergic cells (Sussel et al., 1999; Du et al., 2008).

Developmental cell specification is an area of great current interest for uncovering how neurons are able to integrate information in a highly complex neural circuit (Tasic et al., 2018; for review, see Fishell and Heintz, 2013). The interplay between the expression of specific genetic factors and neuronal activity are essential contributors to the establishment of cell identity (Dehorter et al., 2015; Jabaudon, 2017) and function (for review, see Yap and Greenberg, 2018). Therefore, the concept of specification leads to diversity among neuronal populations and acts as an important foundation for circuit formation and overall brain function (for review, see Breunig et al., 2011; Dehorter et al., 2017; Wamsley and Fishell, 2017). The cholinergic cell population was believed to be relatively homogenous, despite spanning across various brain regions. However, recent studies have shed light on the diversity within these populations, and the importance of cholinergic subpopulations for brain function. This review article highlights developmental origins of cholinergic neurons and explores recent evidence for genetic, morphological, and electrophysiological diversity of the CINs of the striatum.

## Genetic Diversity of Cholinergic Interneurons

### Cholinergic Cell Diversity Arises From Their Developmental Origin

**Figure 2:**
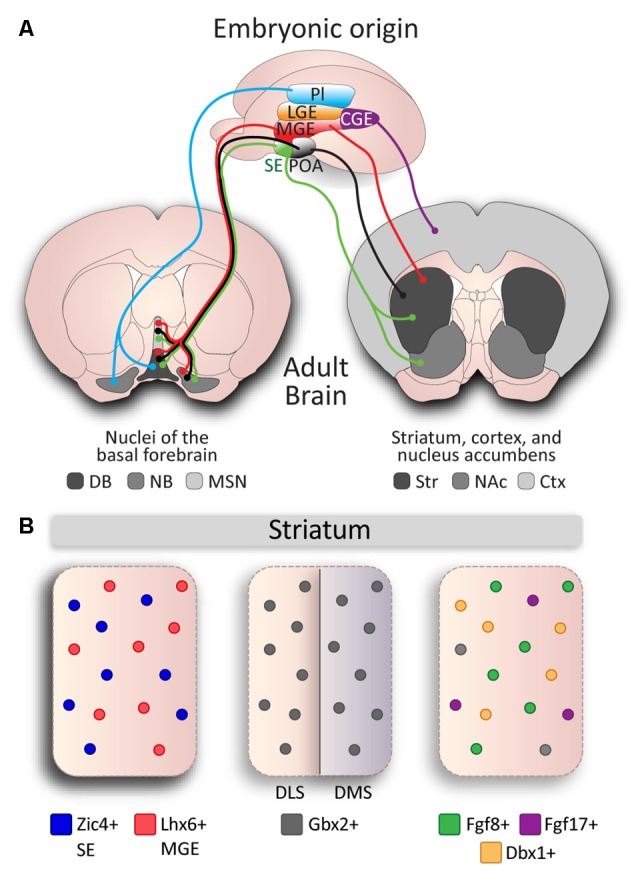
Developmental diversity in cholinergic neurons. **(A)** Cholinergic neurons are derived from different embryonic structures. Proliferative zones of an E13.5 mouse brain embryo: Pallium (Pl), Medial Ganglionic Eminence (MGE), Lateral Ganglionic Eminence (LGE), Septal Epithelium (SE), Preoptic Area (POA), and Caudal Ganglionic Eminence (CGE) give rise to cholinergic neurons of different nuclei: Striatum (Str), Cerebral Cortex (Ctx), Nucleus Accumbens (NAc), and the Basal Forebrain: Nucleus Basalis (NB), Medial Septal Nucleus (MSN), Diagonal Band (DB). **(B)** Genetic diversity in cholinergic interneurons (CINs) in the striatum. Subsets of CINs express Zic4 (blue) and originate from the SE, and Lhx6 (red) and originate from the MGE. Gbx2 is expressed in almost all CINs and preferentially controls development of late-born CINs in the dorsomedial striatum (DMS), but not in early-born CINs in the dorsolateral striatum (DLS). MGE-derived Fgf8 (green) and Fgf17 (purple), and POA-derived Dbx1 (orange) mark three subgroups defined by genetic expression and reliant on developmental origin, but do not account for the entire CIN population (gray).

It is reasonable to expect that cholinergic neurons in a single structure would originate and migrate from a single developmental zone. However, VIP-positive interneurons in the cortex are the only cholinergic population thought to originate from a single developmental proliferative zone, the caudal ganglionic eminence (CGE; Vogt et al., 2014). Cholinergic neurons within a single structure can arise from multiple developmental origins (Nóbrega-Pereira et al., 2010), and conversely, these proliferative areas can also give rise to cholinergic neurons fated to multiple structures in the brain (Marin et al., 2000; Pombero et al., 2011; Magno et al., 2017; Figure 2A). Most basal forebrain cholinergic neurons originate from the MGE, POA (for review, see Ballinger et al., 2016), and SE (Magno et al., 2017). Furthermore, subsets of neurons in the diagonal band and NB originate from the ventral area of the pallial telencephalon (pallium), which is located adjacent to the pallium/subpallium border (Pombero et al., 2011; Ceci et al., 2012; Figure 2A). Similarly, striatal CINs originate from the MGE and POA (Marin et al., 2000; Fragkouli et al., 2009), as well as the SE (Magno et al., 2017). Therefore, to fully appreciate the diversity within cholinergic populations, their developmental origins must be considered. However, as many progenitor regions have genetically defined sub-domains (Flames et al., 2007), neurons originating in the same region may display further genetic diversity.

### Cholinergic Cell Diversity Is Controlled by Genetic Factors

Combinatorial gene expression during early developmental stages can translate to diversity within the mature striatum (Flames et al., 2007). For example, the Dbx1 transcription factor defines a sub-domain of the POA which gives rise to a subset of cholinergic striatal interneurons (Gelman et al., 2011). Similarly, fibroblast growth factor 8 (Fgf8) and fibroblast growth factor 17 (Fgf17) expressing progenitors in the ventral MGE and ventral SE give rise to individual subsets of CINs in the striatum (Hoch et al., 2015a).

A number of studies have also investigated the effect of gene expression on cholinergic cell specification and identity. The transcription factors Lhx8 and Isl1 drive cholinergic identity in MGE/POA-derived neurons that are fated to populate the basal forebrain and striatum (Zhao et al., 2003; Cho et al., 2014). The ablation of either of these genes results in a loss of most cholinergic neurons in the NB, diagonal band and septum (Zhao et al., 2003; Mori et al., 2004; Elshatory and Gan, 2008; Cho et al., 2014). Moreover, it has been shown that Isl1 is required for the specification of a subset of Nkx2.1+/Lhx8+ interneurons, as Isl1 ablation leads to a ~40% of the Nkx2.1-expressing cells in the striatum (Cho et al., 2014). This partial loss of cholinergic cells suggests that the basal forebrain and striatal neuroepithelium contain Lhx8/Isl1-independent cholinergic fated cells. While the basal forebrain is divided into different nuclei, striatal CINs are spread throughout the striatum and were long considered as a single homogeneous population. However, studies into specific genetic factors have shown the diverse origins of CINs in the striatum. For instance, the expression of the transcription factor Zic4 in the SE defines septal cholinergic neurons but also 50% of the striatal CINs, which migrate from the SE (Magno et al., 2017). On the other hand, expression of the Otx2 transcription factor regulates the development of cholinergic neurons derived from the MGE and POA, and the specific ablation of the Otx2 gene from the Nkx2.1 domain results in a loss of some cholinergic neurons of the basal ganglia (Hoch et al., 2015b). It was previously thought that striatal cholinergic cell identity was exclusively defined by the upregulation of Lhx8 and coordinated downregulation of Lhx6 transcription factor (Fragkouli et al., 2009; Lopes et al., 2012), a developmental marker of MGE-derived striatal interneurons (Liodis et al., 2007). However, a subset of CINs was recently found to maintain Lhx6 expression alongside cholinergic markers, resulting in Lhx6 positive (53%) and negative (47%) populations in the striatum (Lozovaya et al., 2018). In addition, a proportion of striatal neurons express the Er81 transcription factor (Dehorter et al., 2015), and these are likely to also be derivatives of Er81-positive progenitor domains from the MGE/POA (Flames et al., 2007).

Another factor to consider to fully appreciate cholinergic cell specification is that during development, neurons acquire different identities according to their time of birth (Kao and Lee, 2010). CINs of the striatum are among the earliest cells born (between E12 and E15) along the caudal-rostral gradient respectively (Semba et al., 1988). Early- and late-born CINs migrate at different time points (Marin et al., 2000) and populate lateral and medial regions of the striatum, respectively (Chen et al., 2010). The Gbx2 transcription factor is implicated in the distinction between these early- and late-born cells and is expressed in almost all CINs. The absence of Gbx2 almost entirely ablates the late-born population (Chen et al., 2010; Figure 2B), and thus may also mark a cholinergic subpopulation in the striatum. These studies demonstrate the presence of diverse genetic patterning during development that is required for the control of cholinergic cell identity. The interactions and overlaying domains of these subgroups has yet to be determined; we are unaware whether they are discrete cholinergic populations or if they arise from combinatorial gene expression that leads to further diversity. In addition, our understanding of the developmental mechanisms behind the regulation of these crucial genetic factors is still limited.

### Advances in Technology Point to Even More Genetic Diversity Within Populations Than First Imagined

An important advancement has been the introduction of single-cell sequencing of neuronal populations to identify patterns of genetic expression (Liu et al., 2018; Zeisel et al., 2018). Using this technique, a number of studies have found substantial genetic diversity among cholinergic neuron populations. For example, the neurochemical patterning of hypothalamic cholinergic neurons in the arcuate nucleus is heterogeneous. These cells show clustering of certain genes coding for POMC-derived peptides, enzymes responsible for the synthesis and release of GABA, glutamate and catecholamines. This heterogeneity has been proposed to be linked to the function of the arcuate nucleus in regulating feeding behavior. Indeed a subset of these cholinergic neurons was found to express leptin and insulin receptors alongside their downstream targets, indicating their specificity (Jeong et al., 2016). In addition, cortical VIP cholinergic neurons also appear genetically diverse when analyzed on a single-cell basis (Zeisel et al., 2015; Tasic et al., 2016). Striatal CINs present diverse genetic patterning and this even extends to cholinergic markers. Individual CINs express different levels of ChAT and choline transporters, such as vesicular acetylcholine transporter (VAChT) and the high-affinity choline transporter (ChT; Muñoz-Manchado et al., 2018). Such genetic variation points to subsequent functional heterogeneity.

## Genetic Diversity May Lead to Functional Subgroups in Cholinergic Neuron Populations

**Figure 3:**
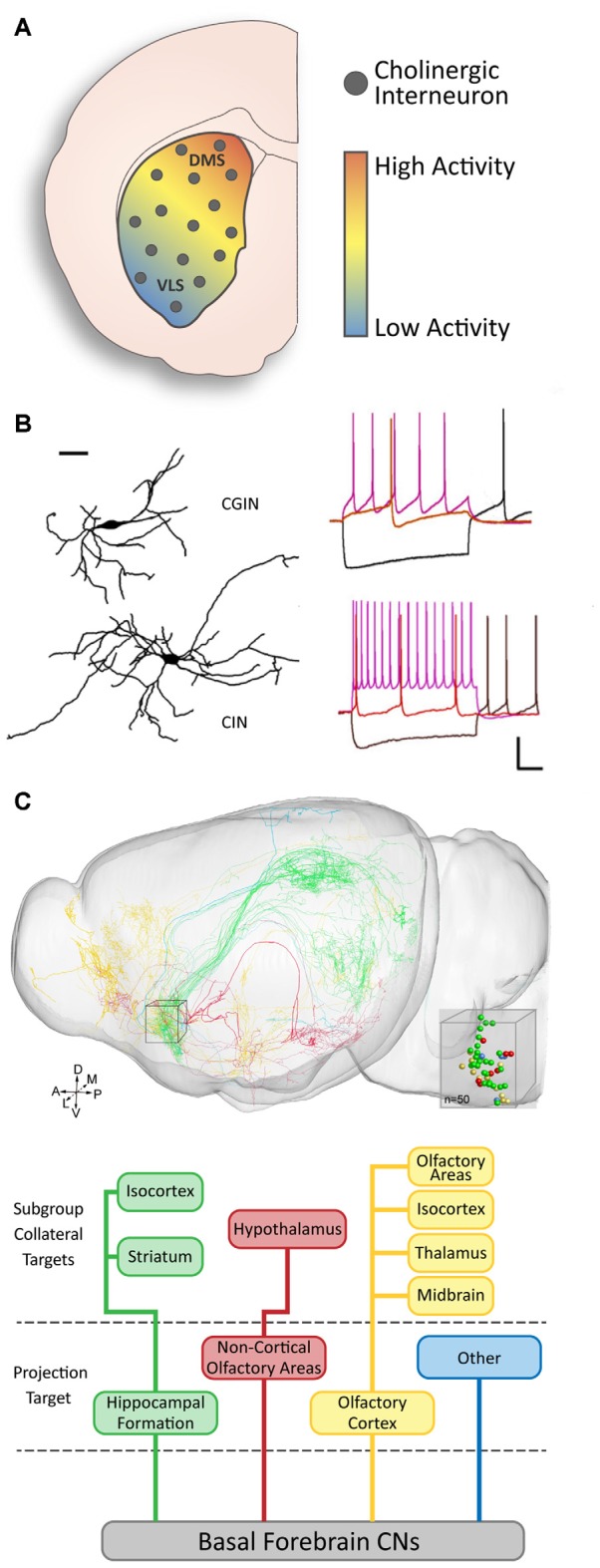
Functional diversity of cholinergic neurons. **(A)** There is a gradient of baseline activity in striatal CINs along the DMS to ventrolateral (VLS) axis (modified from Matamales et al., 2016 with permission).** (B)** Two subsets of striatal CINs show morphological and electrophysiological differences. GABAergic CINs (GCIN) have smaller dendritic fields and slower firing rates than purely cholinergic CINs (adapted from Lozovaya et al., 2018 with permission). **(C)** Basal forebrain cholinergic neurons (CNs) have diverse projection targets and can be grouped into four major categories with subcategories based on collateral targets (adapted from Li et al., 2018 with permission).

Genetic diversity in cholinergic populations has consequential influences on their functional properties and integration within neuronal networks. For instance, there is documented diversity in cholinergic striatal activity, with higher levels of CIN baseline activity in the dorsomedial part compared to the ventrolateral part of the striatum (Figure 3A). The presence of a gradient of the phosphorylated ribosomal protein S6 (p-rpS6), a specific marker of cholinergic activity across the striatum (Matamales et al., 2016; Bertran-Gonzalez et al., 2012), suggests that the regulation of striatal output *via* cholinergic cell activity is heterogeneous within the structure. Several characteristics contribute to the functional features of cholinergic neurons.

### Cholinergic Neurons Are Segregated Into Morphological Subgroups

A number of studies have found evidence for morphological diversity among cholinergic neurons in the brain. In addition to diversity between interneurons and projection neurons in different structures (Figure 1C), diversity within a single population or structure has also been found. For instance, olfactory bulb CINs in the mitral and plexiform cell layers can be segregated into three subsets: locally projecting, bipolar projecting, and non-bipolar projecting (Krosnowski et al., 2012). Similarly, VIP/ChAT-expressing cortical interneurons are also categorized into different morphological subtypes: bipolar, bi-tufted, and multipolar interneurons (Li et al., 2018). However, there is no established genetic or functional correlation for the diversity in these structures. In the striatum, some studies have described a relationship between molecular expression and morphological characteristics in CINs (Figure 3A). In particular, the absence of Gbx2 expression leads to abnormal neurite outgrowth and an increase in the complexity of the dendritic field of CINs (Chen et al., 2010). A correlation between the expression of the Lhx6 transcription factor and CINs morphology has also been recently reported (Lozovaya et al., 2018). The Lhx6-negative CINs present a more complex and vast dendritic arborization, compared to the Lhx6-expressing which are dual cholinergic/GABAergic interneurons (CGINs; Figure 3A). These examples of morphological diversity in cholinergic neurons indicate possible heterogeneity in their connectivity and the role they play within the local and distant circuitry.

### Electrophysiological Properties Differentiate Cholinergic Cell Subtypes

Another critical aspect of circuit integration and formation during development is the electrophysiological properties of cholinergic neurons. In addition to their molecular and morphological characteristics, several cholinergic cell subtypes have been sorted in various brain structures based on their electrophysiological properties. For instance, basal forebrain cholinergic projection neurons are segregated into two subtypes, early firing and late firing (Unal et al., 2012). The early firing neurons (70%) are more excitable, as well as adaptive and susceptible to a depolarization block. The late firing neurons (30%), whilst less excitable, are able to maintain tonic firing at lower frequencies. As the basal forebrain is made up of four main regions, the functional diversity can be partly attributed to neuron location. For example, cholinergic neurons in the NB have properties consistent with early firing neurons (López-Hernández et al., 2017) whereas cholinergic neurons in the medial septal region have been found to display low frequency spiking properties (Simon et al., 2006).

Cholinergic neurons in the PPN are also functionally diverse and have been categorized into four groups. This is based on the neurons having low threshold spiking, a transient outward potassium current (known as A-current), both of these properties, or none. In addition, it has also been found that cells with A-current can be either early or late firing (Baksa et al., 2019). The diversity in functional properties of cholinergic neurons is important as they are tonically firing and thus different characteristics could lead to different ACh release patterns, altering circuit activity.

In local cholinergic circuitry, the cortical bipolar VIP+/ChAT+ interneurons have been characterized as mostly tonically firing with little to no adaptation, with a subset (approximately 12%) of stuttered firing neurons (von Engelhardt et al., 2007). Similarly, striatal CINs have also been found to show diversity in their functional properties (Lozovaya et al., 2018). CGINs have a higher sag amplitude following a hyperpolarizing current, a lower frequency of spontaneous tonic firing, and a more prominent pause response than the CINs. This functional cell diversity implies a fine control of information processing from cholinergic neuron subtypes.

### Heterogeneity in Connectivity Underlies Cholinergic Neuron Diversity

Cholinergic neurons have the ability to modulate the activity of other neurons, therefore, facilitating higher-order processing in numerous nuclei (for review, see Teles-Grilo Ruivo and Mellor, 2013). Their specific and extensive pattern of connectivity is fundamental to their role, yet is different between different populations. The cholinergic projection neurons in the basal forebrain innervate various cortical and subcortical regions. Their organization into subgroups is defined by the cortical region that they target (Zaborszky et al., 2015); whereas the heterogeneity of their outputs is reciprocated in the inputs they received from different areas (Zheng et al., 2018; for review, see Záborszky et al., 2018). It was recently found that the connectivity pattern of cholinergic neurons can be even more complex. For example, within a single region of the basal forebrain, cholinergic neurons can be segregated by their main target region as well as the target locations of their collaterals (Li et al., 2018; Figure 3C). However, we still do not have a full understanding of the intricacies that underpin this system.

It is known that the cortical and thalamic innervation of the striatum follows an approximate topographical gradient, whereby the dorsomedial striatum (DMS) and dorsolateral striatum (DLS) receive inputs from different cortical, thalamic, and brainstem regions (McGeorge and Faull, 1989; Berendse et al., 1992; Bolam et al., 2000; Dautan et al., 2014; Assous et al., 2019). In addition, the dorsal striatum receives more excitatory innervation than the ventral striatum (Assous et al., 2019), indicating that neuronal activity in these regions may be modulated differently. These trends may also extend to influencing CINs, as they receive afferents from the cortex and thalamus (Doig et al., 2014; Klug et al., 2018). DMS CINs have higher levels of activity than those in the DLS (Matamales et al., 2016; Figure 3A).

In addition, a pathway from the PPN to the striatum has been reported, and interestingly consists of cholinergic and glutamatergic afferents that target striatal CINs (Dautan et al., 2014, 2016; Assous et al., 2019). Similarly, inputs from the parafascicular nucleus to the striatum form a topographic map and have also been shown to target CINs (Mandelbaum et al., 2019). Whilst extra-striatal sources of innervation onto CINs have been determined, whether they differently target and influence CIN subtypes is not known.

Striatal CINs are also heavily innervated by the local striatal network. They receive various inhibitory inputs from GABAergic neuron types (English et al., 2011; Straub et al., 2016), as well as the projecting SPNs (for review, see Abudukeyoumu et al., 2019). Moreover, CINs receive dense synaptic inputs from other CINs, forming microcircuitry that then modulates the overall striatal output *via* dense innervation of the spiny projection neurons (English et al., 2011; Straub et al., 2016; Abudukeyoumu et al., 2019). CINs virtually target all cells within the striatum (for review, see Lim et al., 2014). They primarily send connections to neuropeptide Y-expressing interneurons, projection neurons, weak inputs to parvalbumin-expressing interneurons (English et al., 2011; Straub et al., 2016) and likely target tyrosine hydroxylase-expressing interneurons (for review, see Tepper et al., 2018). The assortment of inputs and outputs of CINs may be reliant on their diverse identity. In conclusion, this aspect of striatal network connectivity is yet to be investigated and must be taken into account for future studies of the connectivity pattern of cholinergic cell subtypes.

Despite cholinergic neurons being primarily characterized by their release of Ach, there is a great deal of evidence supporting that some cell subtypes have the ability to co-release different neurotransmitters. For instance, cholinergic neurons from the basal forebrain (Takács et al., 2018) and the medial septum co-release gamma-Aminobutyric acid (GABA) and ACh (Desikan et al., 2018) and express synthesis enzymes for both neurotransmitters (Saunders et al., 2015; Granger et al., 2016). GABAergic identity has also been reported in cortical CINs through their co-expression of glutamic acid decarboxylase (GAD) and cholinergic marker ChAT (von Engelhardt et al., 2007), as expected due to their initial characterization as inhibitory interneurons. Striatal CINs with GABAergic identity defined by Lhx6 expression co-release GABA alongside ACh (Lozovaya et al., 2018). On the other hand, the expression of a vesicular glutamate transporter VGluT3 has been shown in cholinergic axon terminals in the striatum (Gras et al., 2008; Higley et al., 2011; Nelson et al., 2014), implying the potential for co-release of Glutamate and ACh in all striatal CINs. If co-release mechanisms are shown to have a functional influence, they must be regulated to maintain normal circuit activity and may have a role in pathology.

## Discussion

### Cholinergic Cell Diversity Is Established During Development

To understand the organizational logic of cholinergic neurons in circuitry, it is necessary to decipher the biological basis of their cellular diversity. As we have reviewed, the genetic, spatial, and temporal framework of cholinergic cell development provides a foundation for discovering and classifying subpopulations. There have been challenges to the exploration of cell specification due to technological limitations, however, the development of new techniques in the field of molecular genetics (single-cell RNA sequencing, patch-sequencing, optogenetics, and pharmacogenetics) has enabled the exposure of hidden neuronal diversity within cholinergic populations (Fuzik et al., 2016; Liu et al., 2018; Muñoz-Manchado et al., 2018).

It is well understood that the maturation of neuronal circuits requires tightly controlled gene expression cascades (Dehorter et al., 2012; Novak et al., 2013). The function and co-expression pattern of different markers that segregate cholinergic cell populations remain ambiguous. In particular, the role of Er81 (Dehorter et al., 2015) and Zic4 proteins (Magno et al., 2017) in striatal-fated CINs are currently unknown (Figure 2B). Cholinergic cell diversity is a consequence of different embryonic origins and exposure to transcriptional programs during maturation. As we have outlined, seemingly uniform populations of cholinergic neurons having common properties can be derived from multiple embryonic structures (Figure 2A), with unique chemical and morphological traits arising at distinct time points during development. It is likely that chemically, spatially and temporally-restricted domains give rise to unique neuronal populations. Since MGE-derived neurons are generated at different embryonic stages (Mi et al., 2018; Sandberg et al., 2018) and CINs proliferate along a caudal-rostral axis (Semba et al., 1988), future research may reveal which proliferation domains generate cholinergic cell subtypes. Subsequent to proliferation, cells use different migratory pathways that require the expression of specific receptors to detect chemoattractive and chemorepulsive cues (Wichterle et al., 2003; Gelman et al., 2012; Villar-Cerviño et al., 2015; Touzot et al., 2016). Therefore, development of cholinergic neurons that requires the accurate modulation of distinct transcriptional programs needs to be further investigated.

As the complex mechanisms behind neuronal development continue to be revealed, the vital role of activity-dependent processes is central to our understanding. These processes seem to be present throughout the developing brain, however, the transcriptional programs appear to be unique to specific neuronal cell-types (for review, see Dehorter et al., 2017; Yap and Greenberg, 2018). Similar mechanisms are also present in adulthood, modulating the output of neuronal circuitry (Dehorter et al., 2015). If we consider this in context of recently discovered striatal cholinergic subpopulations (Magno et al., 2017; Lozovaya et al., 2018), it is possible that different activity-induced processes tune cholinergic activity in specific ways (Krishnaswamy and Cooper, 2009), delineating further subtypes. However, research into the activity-dependent homeostatic specification of cholinergic neurons are, to the best of our knowledge, very limited (Borodinsky et al., 2004). As a result, studies into this field promise to highlight the fundamental mechanisms of cholinergic cell function.

### Functional Complexity of Neuronal Networks Depends on Cholinergic Cell Diversity

Complex neuronal networks are necessary for higher-order processing and require various cell types and connections. We must consider that genetically-diverse CINs may fit into the striatal network in unique ways. For instance, Lhx6-positive and negative CINs in the striatum display functional differences (Lozovaya et al., 2018). Although they share several major common intrinsic membrane properties, Lhx6-positive CINs display higher sag amplitudes in response to hyperpolarizing pulses than Lhx6-negative CINs, lower spontaneous spiking frequency, and smaller dendritic arbors (Figure 3B). Therefore, there is the possibility that they form different connections, and integrate differently in the network.

The striatum has neurochemically diverse regions that are formed during development and may contribute to the formation of subnetworks. The striosome and matrix regions are three-dimensional, interlocked structures constituting neurochemically and morphologically distinct domains, which emerge during development and also have an important role in the mature striatum (Hagimoto et al., 2017; for review, see Brimblecombe and Cragg, 2017). Evidence has shown that early-born striatal cells migrate to striosomes and connect preferentially with limbic circuits, whereas late-born cells populate the matrix and receive dominant input from the neocortex (Crittenden et al., 2017; Hamasaki and Goto, 2019). In addition, CIN dendritic fields preferentially occupy the matrix compartments, alongside higher levels of cholinergic markers, suggesting that cholinergic neurons development is also dependent on the striosome-matrix structure (Crittenden et al., 2017). However, the correlation between CIN diversity and the compartmentalized structure of the striatum remains to be explored.

As we have described, cholinergic neurons can present different morphological and electrophysiological properties (Figure 1B). They are known to innervate other structures with different patterns of connectivity (Li et al., 2018; Figure 3C), and also receive inputs from various structures and cell types (for review, see Lim et al., 2014). It is thus crucial to determine whether cholinergic cell subtypes exhibit different morphological features (i.e., axonal and dendritic fields) or innervation patterns to ultimately fully characterize their involvement in various microcircuits.

Cholinergic transmission can mediate disparate actions *via* integration of postsynaptic signals (Calabresi et al., 2000). It is necessary to consider the function and distribution of postsynaptic receptors that are scattered across various cells and receptors with different signaling capacities that can be co-expressed in the same cell type. Increased understanding of the specific neuron types responsive to ACh and their functional connectivity is necessary to obtain a phenomenological understanding of neuromodulation and behavior both at the cellular and circuit levels. Interplay between ACh and other neurotransmitters are central for basal ganglia function (for review, see Bonsi et al., 2011), and ACh-dopamine (DA) coupling has been extensively studied, as their reciprocal interaction largely contributes to the control of the striatal output. For example, DA has a fundamental role in the striatum (Tritsch et al., 2012; for review, see Cools, 2011), as it controls the pause in CIN firing (Zhang et al., 2018), both in the medial and lateral areas. There are regional differences in the dopaminergic control of striatal CINs. It has been described that the pause of these cells in the DLS is shorter than in the DMS, *via* a mechanism of DA-glutamate co-transmission (Chuhma et al., 2018). Moreover, activation of glutamatergic receptors by cortical and thalamic inputs might lead to distinct integration strategies from CINs subtypes in the striatum (Kosillo et al., 2016).

CINs are fundamental to the integration of somatosensory information and motor-related signals (for review, see Robbe, 2018). There is a functional dissociation between the DMS, which is responsible for goal-directed actions, and the DLS, supporting procedural learning such as habit formation (Okada et al., 2018). There are also differences in the sensitivity of tonically active neurons to rewarding events between dorsal and ventral striatum (Marche et al., 2017). Determining whether responses of cholinergic cell types vary is an interesting topic for future studies utilizing *in vivo* optogenetics, calcium imaging or electrophysiological approaches.

Our understanding of the role of cholinergic subpopulations within pathological conditions is currently limited, however, a study reported unique roles of striatal CINs and GABA/ACh co-expressing striatal interneurons (CGINs; Figure 3B) in PD (Lozovaya et al., 2018). It shows that DA deprivation specifically strengthens CGIN-CGIN network and abolishes both GABAergic inhibition and pause response in CGINs. Future studies should focus on distinguishing between the different cholinergic subpopulations on the basis of their embryonic origin, molecular profile and connectivity, when analyzing cholinergic network dysfunction in disease conditions.

## Conclusion

Despite significant research into cholinergic neurons, the recent discovery of diverse subpopulations has reinforced the notion that our understanding of these cells remains incomplete. In this review, we have combined the current literature surrounding cholinergic neurons in the brain with a specific focus on the newfound diversity within cholinergic population of the striatum. Whereas similarities between cholinergic populations predicated that neurons were relatively functionally homogenous, studies show genetic, morphological and electrophysiological properties in striatal cholinergic subpopulations. This diversity likely underpins their specific function in normal and pathological conditions. Future studies into cholinergic cell subpopulations aiming at understanding their development, morphology and activity are necessary to ultimately determine the implications of this new-found cell diversity in health and disease.

## Author Contributions

All authors contributed to the writing of the manuscript, and all have approved it for publication.

## Conflict of Interest Statement

The authors declare that the research was conducted in the absence of any commercial or financial relationships that could be construed as a potential conflict of interest.
